# The *Arabidopsis* transcription factor IIB-related protein BRP4 is involved in the regulation of mitotic cell-cycle progression during male gametogenesis

**DOI:** 10.1093/jxb/eru140

**Published:** 2014-04-10

**Authors:** Zhixiang Qin, Xiaoran Zhang, Xiao Zhang, Wei Xin, Jia Li, Yuxin Hu

**Affiliations:** ^1^Key Laboratory of Plant Molecular Physiology, Institute of Botany, Chinese Academy of Sciences, Beijing 100093, PR China; ^2^University of Chinese Academy of Sciences, Beijing, PR China; ^3^School of Life Sciences, Lanzhou University, Lanzhou, PR China; ^4^National Center for Plant Gene Research, Beijing, PR China

**Keywords:** *Arabidopsis*, BRP4, male gametogenesis, mitosis, ORC6, TFIIB-related protein.

## Abstract

Male gametogenesis in angiosperms involves two rounds of mitosis that are essential for the generation of two sperm cells to achieve double fertilization, a distinct event in the sexual reproduction of flowering plants. Precise regulation of mitosis during male gametogenesis is critically important for the establishment of the male germline. However, the molecular mechanisms underlying mitotic division during male gametophyte development have not been characterized fully. Here, we report that the *Arabidopsis* transcription initiation factor TFIIB-related protein BRP4 is involved in the regulation of mitotic cell-cycle progression during male gametogenesis. *BRP4* was expressed predominately in developing male gametophytes. Knockdown expression of *BRP4* by a native promoter-driven RNA interference construct in *Arabidopsis* resulted in arrest of the mitotic progression of male gametophytes, leading to a defect in pollen development. Moreover, we showed that the level of expression of a gene encoding a subunit of the origin recognition complex, *ORC6*, was decreased in *BRP4* knockdown plants, and that the *ORC6* knockdown transgenic plants phenocopied the male gametophyte defect observed in *BRP4* knockdown plants, suggesting that *ORC6* acts downstream of *BRP4* to mediate male mitotic progression. Taken together, our results reveal that *BRP4* plays an important role in the regulation of mitotic cell-cycle progression during male gametogenesis.

## Summary

This work revealed that the *Arabidopsis* TFIIB-related protein BRP4 is critical for male gametogenesis by modulating mitotic cell-cycle progression.

## Introduction

The formation and development of male gametophytes in flowering plants involve both meiosis and mitosis. These processes generate a pair of functional sperm cells that are required for the success of subsequent double fertilization, an event specific and essential for plant sexual reproduction (McCormick, 1993; Berger and Twell, 2011). Male gametophytes of plants come from the pollen mother cells, which undergo a round of meiosis to produce a tetrad of microspores that are then released within reproductive locules. The uninucleate microspores undergo two rounds of mitotic cell division, which occur either before or after pollination, depending on the plant species (McCormick, 1993). The first mitotic cell division, pollen mitosis I (PMI), occurs asymmetrically to produce two differently sized cells with different fates (McCormick, 1993; Berger and Twell, 2011). The larger vegetative cell arrests at G1 phase and exits from the cell cycle, while the smaller generative cell undergoes a second symmetric mitosis, pollen mitosis II (PMII), and produces two sperm cells that are delivered to the female ovule via pollen tubes for double fertilization (Tanaka, 1997; Twell *et al.*, 1998; Borg *et al.*, 2009). Using both genetic and molecular genetic approaches, some of the key genes involved in microspore formation have been identified in *Arabidopsis*. Furthermore, the regulatory network underlying early anther development has been characterized (Ma *et al.*, 2012). By contrast, our knowledge of the genetic and molecular regulation of the mitotic cell-cycle progression during male gametogenesis is still limited.

DUO POLLEN1 (DUO1) was the first transcription factor shown to be expressed specifically in generative cells. The microspores of *duo1* plants can go through PMI, but the generative cells cannot undergo PMII. This leads to the formation of bicellular-like pollen grains that are incapable of finishing double fertilization (Durbarry *et al.*, 2005; Rotman *et al.*, 2005). DUO1 has been shown to regulate three germline marker genes, *Histone H3.3 Variant* (*MGH3/HRT10*), *GAMETE EXPRESSED2* (*GEX2*), and *GENERATIVE-CELL SPECIFIC1* (*GCS1/HAP2*) (Brownfield *et al.*, 2009a). Recently, several additional genes, such as *DUO1-ACTIVATED TARGET* (*DAT*), were found possibly to be regulated by *DUO1* (Borg *et al.*, 2011). DUO POLLEN3 (DUO3) is another regulator whose function is similar to DUO1. DUO3 coordinates germ-cell division with DUO1, and may share the same regulatory targets as DUO1, as DUO3 is known to be required for the expression of *GEX2* and *GCS1*/*HAP2* (Brownfield *et al.*, 2009b). The A-type cyclin-dependent kinase;1 (CDKA;1), a key regulator of both the G1/S and the G2/M phase transitions in the cell-cycle progression, was shown to be required for entry into generative-cell division, as *cdka;1* produces bicellular pollen containing a single sperm-like cell and a vegetative cell (Iwakawa *et al.*, 2006). Similarly, mutation of an F-box protein (FBL17) also causes a pollen defect similar to that observed for the *cdka;1* mutant. FBL17 forms an SCF-type complex with the SKP1-like protein11 (ASK11) to target Kip-related protein 6 (KRP6) and KRP7 for proteasome-dependent degradation (Kim *et al.*, 2008; Gusti *et al.*, 2009). In addition, disruption of the *Aberrant Pollen Development1* (*APD1*) and *APD2* genes, which encode plant-specific RING-finger proteins, increases the percentage of bicellular-like pollen at the mature pollen stage; this phenotype is enhanced by downregulation of *APD3* or *APD4* (Luo *et al.*, 2012). Recent studies in *Arabidopsis* have also identified a few genes that participate in the regulation of PMI. For example, simultaneous mutation in *RING-H2 group F1a* (*RHF1a*) and *RHF2a* not only leads to a defect in pollen development mainly due to the arrest of PMI but also blocks mitotic cell division during female gametophyte formation (Liu *et al.*, 2008). This result indicates that these genes function in both male and female gametogenesis. RHF1a was also shown to interact directly with and target a cyclin-dependent kinase inhibitor, ICK4/KRP6, for proteasome-mediated degradation (Liu *et al.*, 2008).

The initiation of eukaryotic gene transcription requires the formation of a pre-initiation complex (PIC) on promoter regions. The PIC is composed of PolII, transcription factor IIB (TFIIB), and TATA box-binding proteins (TBPs) (Roeder, 1996; Kostrewa *et al.*, 2009). TFIIB transcription factors are central components of these complexes, as they not only recruit PolII to the promoter but also bind DNA and TBPs (Nikolov *et al.*, 1995; Kostrewa *et al.*, 2009). The crystal structure of the PolII-B complex shows that a zinc ribbon domain (B-ribbon) at the N terminus of TFIIB proteins can bind PolII, while its C-terminal domain (B-core) binds DNA and TBP (Kostrewa *et al.*, 2009). In *Arabidopsis*, there are 14 TFIIB-like proteins that have been phylogenetically categorized into the TFIIB, Brf, and Rrn7/TAF1B/MEE12 subfamilies (Knutson, 2013; Niu *et al.*, 2013). The TFIIB subfamily has eight members, including TFIIB1, TFIIB2, and six BRP proteins, among which BRP3 and BRP6 contain only partial TFIIB domains and are predicted to function differently from the other TFIIBs (Knutson, 2013). A recent study revealed that *TFIIB1* plays important roles in pollen-tube growth and endosperm development, as mutation in *TFIIB1* results in retarded growth of the pollen tube, impaired pollen-tube guidance and reception, and abnormal endosperm development (Zhou *et al.*, 2013). It is interesting that the defective pollen-tube growth and guidance and reception can be restored completely by expression of *TFIIB2* driven by the *TFIIB1* promoter, and the defective seed development phenotype can be rescued by the expression of *TFIIB3*/*BRP2* driven by the *TFIIB1* promoter. These results suggest that these three genes function in a partially redundant and cooperative manner (Zhou *et al.*, 2013)*. BRP1* is expressed ubiquitously and encodes a plant-specific TFIIB-related protein that is localized both to plastids and to the nucleus (Lagrange *et al.*, 2003). BRP1 has been reported to be involved in RNA polymerase I-dependent rRNA synthesis (Imamura *et al.*, 2008). By contrast, the expression of *BRP2* is restricted to reproductive organs and seeds, and *BRP2* is involved in the regulation of endosperm growth as *brp2* exhibits a slower proliferation rate at the endosperm syncytial stage (Cavel *et al.*, 2011). *BRP5/PTF2* also encodes a plant-specific TFIIB-related protein and participates in pollen germination and embryogenesis, and its expression is abundant in developing pollen, embryos, and shoot apical meristems (Niu *et al.*, 2013).

Here, we report that *Arabidopsis* transcription initiation factor BRP4, another member of the TFIIB-related protein family, is involved in the regulation of mitotic cell-cycle progression during male gametogenesis. *BRP4* was highly expressed in developing male gametophytes, with a peak in expression at the tetrad stage of microspore development. Knockdown of *BRP4* expression by a native promoter-driven RNA interference (RNAi) construct in *Arabidopsis* predominantly aborted male gametophyte development by arrest of the male gametophyte mitotic cell-cycle progression. We have provided evidence that a gene encoding a subunit of the origin recognition complex, *ORC6*, may act downstream of *BRP4*, and showed that *ORC6* is required for male gametophyte cell-cycle progression. These findings suggest that BRP4-mediated male gametogenesis may function, at least in part, through ORC6-regulated cell division machinery.

## Materials and methods

### Plant materials and growth conditions

*Arabidopsis thaliana* accession Columbia-0 plants were used in this study, with the exception that the *small organ 3-D* (*smo3-D*) mutant was initially isolated from a WS2 background. All seeds were sterilized in 0.5% sodium hypochlorite for 15min, and then geminated on 1/2 MS medium in a culture room at 22 °C under a 16h light/8h dark photoperiod with an illumination intensity of 80–90 μmol m^–2^ s^–1^. Seven-day-old seedlings were transferred to soil and grown in a growth room at 22±1 °C under the same photoperiod and illumination as those in the culture room (Jing *et al.*, 2009). For morphological characterization of *smo3-D*, 30-d-old plants were used for examination of the leaf area, and 50-d-old plants were used to determine the plant height and silique length.

### Sequence alignment and phylogenetic tree generation

The sequences of TFIIBs and BRP2 were obtained from The Arabidopsis Information Resource (TAIR) database. Alignment analysis was performed with the MUSCLE program (http://www.ebi.ac.uk/Tools/msa/muscle/), and then manually optimized with Genedoc software (Nicholas and Nicholas, 1997; Liu *et al.*, 2013). The full-length amino acid sequences of the BRP homologues in *Arabidopsis* were used for maximum-likelihood phylogenetic analysis. The phylogenetic tree was reconstructed with PHYML version 2.4 using the GTR+I+G model, and 1000 bootstrap replicates were performed (Guindon and Gascuel, 2003; Liu *et al.*, 2013).

### Gene expression analysis

Total RNA was isolated using TRIzol reagent (Invitrogen). RNA was digested with DNase I and reverse transcribed with Superscript III reverse transcriptase (Invitrogen) into cDNA for reverse transcription (RT)-PCR or quantitative (q)RT-PCR analysis. *GLYCERALDEHYDE-3-PHOSPHATE DEHYDROGENASE C SUBUNIT* (*GAPC*) or *ACTIN2* was used as an internal control in the RT-PCR or qRT-PCR analysis, respectively. qRT-PCR analysis was carried out with SYBR Premix Ex Taq Mix on a Rotor-Gene3000 (Corbett Research) by three biological replicates, according to the manufacturer’s instructions. All primers used in the expression analyses are listed in Supplementary Table S1 at *JXB* online.

For the RNA *in situ* hybridization experiments, inﬂorescences were collected from 30 individual 5-d-old plants and fixed in 4% paraformaldehyde. The inflorescences were then embedded in paraffin and sectioned to an 8 μm thickness. A 157bp cDNA fragment specific for *BRP4* was ampliﬁed by PCR to generate digoxigenin-labelled sense and antisense probes. RNA *in situ* hybridization was performed according to a previously described protocol (Chen *et al.*, 2007), and hybridization signals were visualized under a microscope (Olympus BX51).

For the β-glucuronidase (GUS) staining assay, the inflorescences and other organs of homozygous transgenic plants carrying a *proBRP4:GUS* construct were incubated in a 50mM sodium phosphate solution (pH 7.0) containing 5mM K_4Fe_(CN)_6_, 5mM K_3_Fe(CN)_6_, 0.1% Triton X-100, and 1mM 5-bromo-4-chloro-3-indolyl-β-d-glucuronic acid at 37 °C for several hours (Feng *et al.*, 2011).

### Plasmid construction and *Arabidopsis* transformation

To generate the *pro35S:BRP4*, *pro35S:antiBRP4*, *pro35S:BRP2*, and *pro35S:TFIIBs* constructs, the coding sequences of *BRP4*, *TFIIBs*, and *BRP2* were amplified by RT-PCR and ligated into the pEASY-Blunt vector (TransGen Biotech). The plasmids were sequenced for verification and then digested with appropriate restriction endonuclease(s) and cloned into the pVIP96 or pCAMBIA1302 plasmid (Hu *et al.*, 2003). A *BRP4* fragment digested with *Spe*I and *Asc*I was cloned into the pMDC83 plasmid for generation of *pro35S:BRP4-GFP* (Curtis and Grossniklaus, 2003). A *BRP4* fragment digested with *Xho*I and *Spe*I was cloned into the pER10 plasmid to generate the chemically inducible *proXVE:BRP4* construct (Zuo *et al.*, 2000). For the RNAi constructs, a 157bp cDNA fragment specific for *BRP4* or a 210bp fragment from *ORC6* was cloned into the pCAMBIA1300 plasmid containing an RNAi fragment in both the sense and antisense orientations (Qin *et al.*, 2005), and then the appropriate promoter region was cloned into the resulting plasmid to generate *proBRP4:BRP4 RNAi*, *proACT11:BRP4 RNAi*, and *proBRP4:ORC6 RNAi* constructs. For the *proBRP4:GUS* construct, a 2500bp genomic fragment from the *BRP4* promoter was cloned into the pBI101 vector following digestion with *Bam*HI and *Sal*I. All primers used for generation of the constructs are listed in Supplementary Table S2 at *JXB* online.

All constructs were introduced into *Arabidopsis* by *Agrobacterium tumefaciens*-mediated transformation via the floral dip method, as described previously (Clough and Bent, 1998). At least 15 independent lines harbouring a single T-DNA insertion from each construct were generated, and at least three independent lines of their T3 plants were used for detailed characterization.

### Alexander staining and 4′,6-diamidine-2-phenylindole dihydrochloride (DAPI) staining

To observe the developmental status of male gametophyte, anthers were dissected from flowers and mounted on 1/2 MS medium and visualized under a microscope (Olympus BX51). To determine the viability of mature pollen, pollen grains were stained in Alexander’s solution (Alexander, 1969). To visualize nuclei of microspores, stamens at different stages were detached and placed into 0.5 µg µl^–1^ of DAPI staining solution (Park *et al.*, 1998; Liu *et al.*, 2008). The Alexander- and DAPI-stained microspores or pollen grains were photographed, and their developmental status was determined under the microscope.

DAPI fluorescence was used to quantify the relative nuclear DNA content in generative cells. DAPI-stained images were recorded with a fixed exposure and then the DAPI fluorescence values of generative-cell nuclei were determined with ImageJ version 1.4.3.67 software (http://rsb.info.nih.gov/ij/). A net value for each nucleus was calculated after subtraction of the corresponding cytoplasmic background signal. The mean fluorescence value of the generative prophase nuclei from wild-type (WT) bicellular pollen was considered as 2C, and the values obtained were then normalized against this value to calculate the relative C values (Gusti *et al.*, 2009).

## Results

### Ectopic expression of *BRP4* has a pleiotropic effect on organ development

**Fig. 1.**
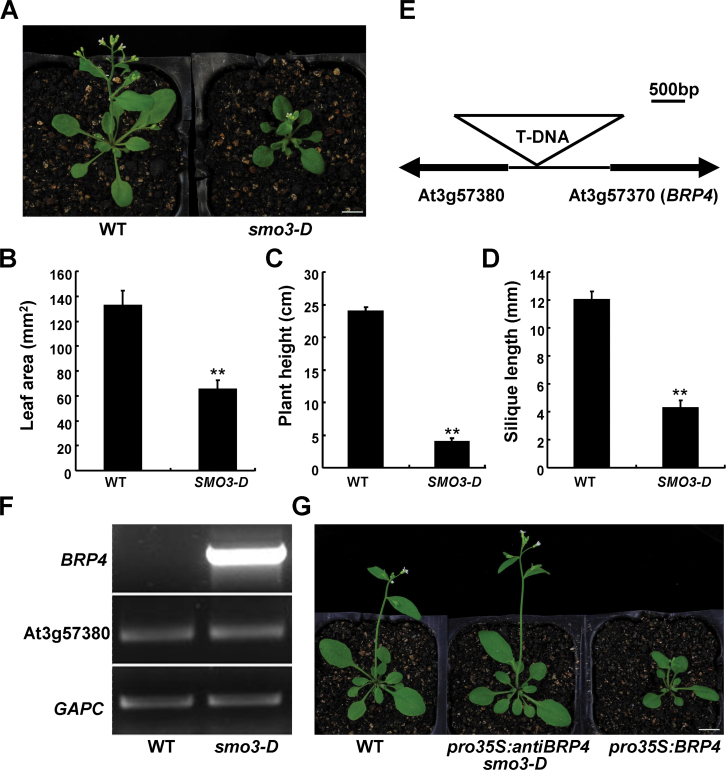
Ectopic expression of *BRP4* affects aerial organ development. (A) Morphology of 23-d-old WT, left) and *smo3-D* plants (right). Bar, 1cm. (B) The area of fully expanded fifth leaves of WT and *smo3-D* plants. At least six leaves from each genotype were used to determine areas, and the data are shown as means±standard deviation (SD) (Student’s *t*-test, **P<0.01). (C, D) Plant height (C) and silique length (D) of WT and *smo3-D* plants. Fifty-day-old plants were used for determination of plant height and silique length; the data were from 15 plants of each genotype and data are presented as means±SD (Student’s *t*-test, **P<0.01). (E) Schematic illustration of the genomic region at the *BRP4* locus in *smo3-D*. The coding regions of both At3g57370 (*BRP4*) and At3g57380 are indicated as black arrows, and the intermediate genomic sequence is indicated as a black line. T-DNA was inserted in the promoter region of *BRP4*, 1014bp upstream of ATG. (F) RT-PCR analysis of *BRP4* and At3g57380 expression in WT and *smo3-D* plants. (G) Morphology of 3-week-old transgenic *pro35S:BRP4* plants and *smo3-D* carrying a *pro35S:antiBRP4* construct. Bar, 1cm.

We initially isolated a dominant mutant, *smo3-D*, from a transgenic *Arabidopsis* population harbouring the T-DNA activation-tagging plasmid pSKI015 (Weigel *et al.*, 2000). The most striking phenotype of *smo3-D* plants was the small size of their aerial organs (Fig. 1A). Compared with that of WT, the size of leaves, inflorescences, and siliques in the *smo3-D* plants was obviously reduced (Fig. 1B–D). Genetic analysis of a backcrossed F2 progeny indicated that the segregation ratio of mutant:WT was 83:28 (3:1, *P*=0.96, χ^*2*^ test), in which all of the mutant phenotypes co-segregated with a T-DNA insertion event. These results suggested that *smo3-D* is a single-gene dominant mutant that may be caused by a T-DNA insertion. To identify the gene responsible for the *smo3-D* phenotype, we amplified the genomic sequence flanking the T-DNA by thermal asymmetric interlaced-PCR (Liu *et al.*, 1995), and found that a T-DNA segment was inserted between At3g57380 and At3g57370 (*BRP4*), 1014bp upstream of the start codon of *BRP4* (Fig. 1E). RT-PCR analysis showed that the expression of *BRP4* was highly upregulated in *smo3-D* plants (Fig. 1F). To examine whether the ectopic expression of *BRP4* was responsible for the small aerial organ phenotype in *smo3-D*, we introduced a *pro35S:BRP4* construct into WT plants and introduced a *pro35S:antiBRP4* construct into *smo3-D* plants. As shown in Fig. 1G, transgenic plants overexpressing *BRP4* also showed the small aerial organ phenotype observed in *smo3-D*, while introduction of the *pro35S:antiBRP4* construct fully restored *smo3-D* to WT morphology. These results indicated that the small aerial organ phenotype in *smo3-D* results from the elevated expression of *BRP4*, and that the ectopic expression of *BRP4* has a pleiotropic effect on organ development.

### BRP4 is a TFIIB-related protein

BLAST searching in the TAIR database showed that the amino acid sequence of BRP4 was highly similar to that of BRP2 (At3g29380), TFIIB1 (At2g41630), and TFIIB2 (At3g10330), all of which belong to the TFIIB family. Alignment indicated that these four proteins shared a conserved zinc ribbon domain and a C-terminal domain with two cyclin folds (Supplementary Fig. S1A at *JXB* online). However, compared with the other three TFIIB members, BRP4 had longer B-finger and B-linker domains (Supplementary Fig. S1A). In *Arabidopsis*, there are 14 TFIIB-like proteins. Phylogenetic analysis showed that TFIIB1, TFIIB2, BRP2, BRP4, BRP6, and BRP3 were clustered into a clade, although BRP4 appeared to be far from the other five members (Knutson, 2013; Niu *et al.*, 2013) (Supplementary Fig. S1B). To test for the possibility of functional redundancy among these TFIIB family members, we overexpressed *TFIIB1*, *TFIIB2*, and *BRP2* in *Arabidopsis*. Compared with the small aerial organs observed in the transgenic plants overexpressing *BRP4*, no morphological phenotypes were observed in transgenic plants overexpressing *TFIIB1*, *TFIIB2*, or *BRP2* (Supplementary Fig. S1C, D), suggesting that *BRP4* might have a unique function in plant development.

### *BRP4* is predominately expressed during the development of male gametophytes

**Fig. 2.**
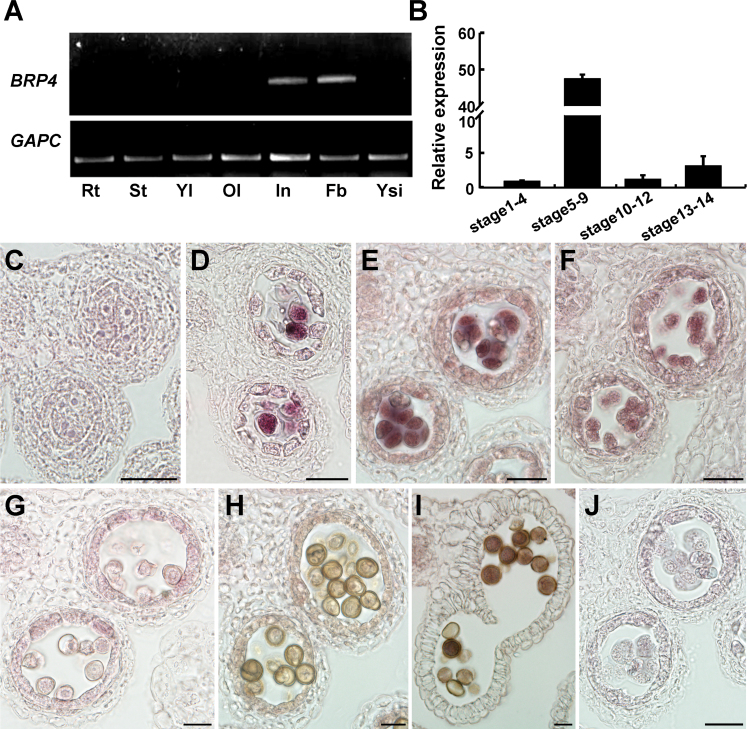
Expression of *BRP4*. (A) RT-PCR analysis of *BRP4* expression in various organs. Rt, root; St, stem; Yl, young leaf; Ol, old leaf; In, inflorescence; Fb, flower bud; Ysi, young silique. (B) Expression of *BRP4* in anthers at the anther different developmental stages assayed by qRT-PCR. The data are from three biological replicates and are presented as means±SD. (C–J) *BRP4* mRNA accumulation assayed by RNA *in situ* hybridization. RNA *in situ* hybridization was performed with anthers at stage 5 (C), stage 6 (D), stage 7 (E), stage 8 (F), stage 9 (G), stage 11 (H), and stage 12 (I). The anther at stage 7 hybridized with a *BRP4* sense probe is shown as an example negative control (J). Bars, 20 µm.

To explore the role of *BRP4* in plant development, we initially examined the organ-specific expression of *BRP4* with RT-PCR analysis and found that *BRP4* transcripts were detected only in the inflorescences and flower buds (Fig. 2A). We then generated transgenic plants carrying a *proBRP4:GUS* construct, and GUS staining assays showed that *BRP4* expression was expressed predominately in developing anthers and mature pollen (Supplementary Fig. S2A, B, at *JXB* online). These observations were consistent with the expression profiles of *BRP4* revealed by the microarray data in the TAIR database (Supplementary Fig. S2C, D). To define the stage-specific expression of *BRP4*, we carried out qRT-PCR analysis with anthers at various developmental stages, and found that *BRP4* was expressed primarily from anther development stages 5–9 (Fig. 2B), during which time the pollen mother cells are undergoing meiosis to produce tetrads and the microspores are then released (Sanders *et al.*, 1999; Ma *et al.*, 2012). Further RNA *in situ* hybridization verified that mRNA of *BRP4* was initially detected at anther development stage 6, when pollen mother cells were undergoing meiotic division (Fig. 2C, D), and that *BRP4* mRNA accumulation reached a peak level in the tetrads at anther development stage 7. *BRP4* mRNA was also detectable in microspores at stage 8 (Fig. 2E, F). However, no *BRP4* mRNA was detected in anthers after stage 9 (Fig. 2G–J). *BRP4* mRNA was also detected in tapetum from anther development stages 5–8 (Fig. 2D–F). These findings strongly suggested that *BRP4* may function during male gametophyte development. Additionally, we examined the cellular localization of BRP4 with *pro35S:BRP4-GFP* transgenic plants, which again showed the small aerial organ phenotype of *pro35S:BRP4* plants (Supplementary Fig. S2E). Green fluorescent protein (GFP) ﬂuorescence of the BRP4–GFP fusion protein in both root and petal epidermal cells showed that BRP4 was localized to the nucleus (Supplementary Fig. S2F, G).

### Knockdown of *BRP4* expression results in pollen developmental defects

**Fig. 3.**
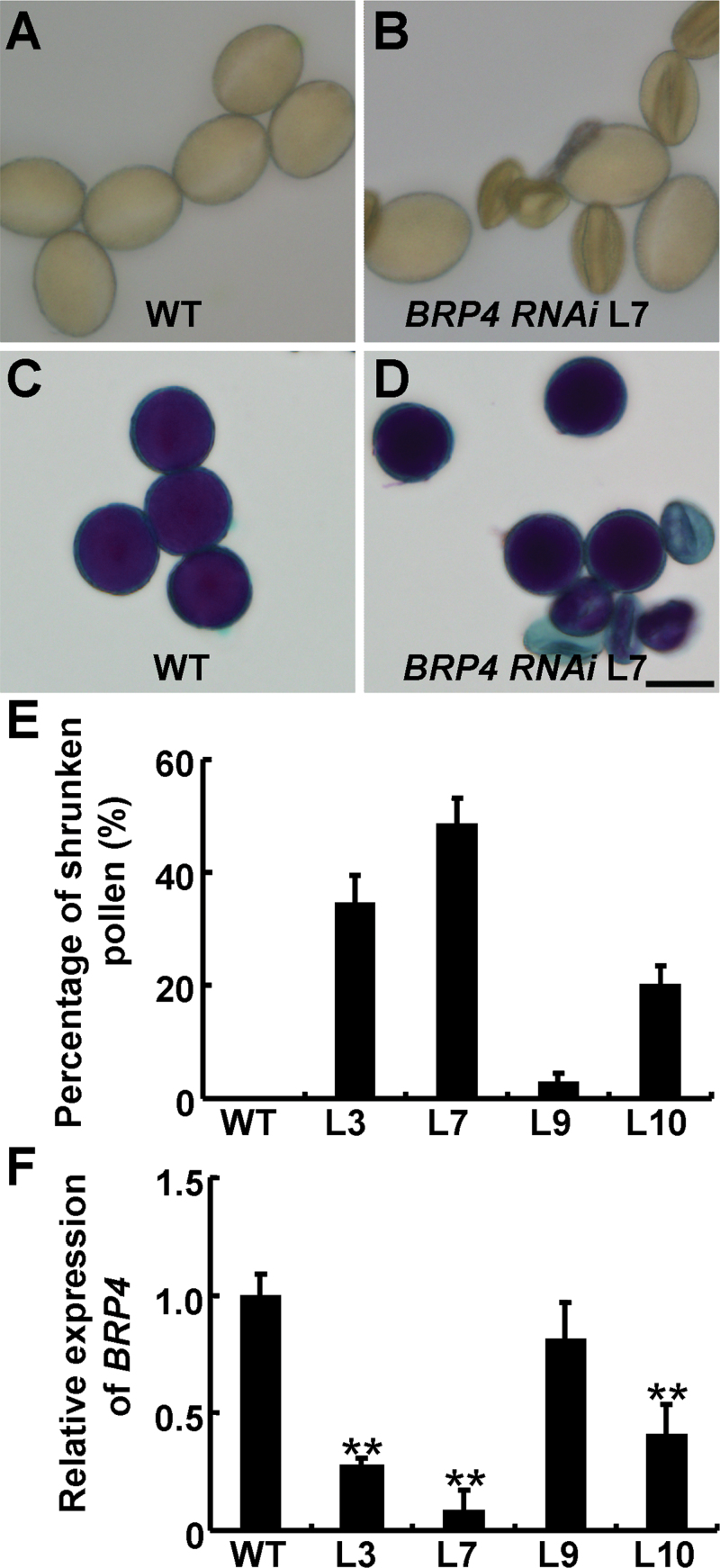
Knockdown of *BRP4* expression causes an aborted pollen phenotype. (A, B) Morphology of mature pollen from WT (A) and transgenic *proBRP4:BRP4 RNAi* L7 plants (B). (C, D) Alexander staining of pollen from WT (C) and L7 (D) plants. Bar, 20 µm. (E) Percentage of the shrunken pollen in WT and four independent T3 transgenic *proBRP4:BRP4 RNAi* lines (L3, L7, L9, and L10). (F) qRT-PCR analysis of the expression of *BRP4* in WT and *proBRP4:BRP4 RNAi* lines as described in (E). The data are from three biological replicates and are presented as means±SD (Student’s *t*-test, **P<0.01).

As no knockdown or knockout mutant of *BRP4* in *Arabidopsis* is publically available, we used an RNAi approach to investigate the biological function of *BRP4*. A native promoter-driven *BRP4* RNAi construct (*proBRP4:BRP4 RNAi*) was generated with a 157bp cDNA fragment specific for *BRP4* and introduced into *Arabidopsis* (Supplementary Fig. S3A at *JXB* online). All of the transgenic plants from the 34 independent *proBRP4:BRP4 RNAi* lines we obtained developed normally in their organ morphology. However, 18 lines displayed a pollen abortion phenotype, with varied percentages of shrunken pollen (Fig. 3A, B). Further analysis of mature pollen in the T3 plants using Alexander staining confirmed the different percentages of abnormal pollen (Fig. 3C, D). Of the four independent lines that were examined closely, L7 produced about 48.64% shrunken pollen, while the percentages of shrunken pollen in L3, L10, and L9 were 34.75, 20.39, and 2.94%, respectively (Fig. 3C–E). qRT-PCR analysis showed that the variations of the aborted pollen phenotype were closely correlated to the reduced *BRP4* expression levels in these lines (Fig. 3E, F). Expression of other *TFIIB* genes closely homologous to *BRP4* was not obviously altered in these *proBRP4:BRP4 RNAi* lines (Supplementary Fig. S3B). These results suggested that the pollen development phenotype in these transgenic plants resulted from the knockdown of *BRP4* expression. In addition, these transgenic plants also exhibited reproductive abnormalities. Compared with WT plants, which produced only 1.04% undeveloped ovules or seeds in siliques, the transgenic lines L7, L3, L10, and L9 had 40.62, 10.06, 3.87, and 1.22% aborted ovules or seeds, respectively (Supplementary Fig. S3C), demonstrating that knockdown of *BRP4* expression also affects plant fertility.

To further verify whether or not the stage-specific knockdown of *BRP4* expression was responsible for the pollen defects in the *proBRP4:BRP4 RNAi* transgenic plants, we also generated transgenic plants carrying a *proACT11:BRP4 RNAi* construct (Supplementary Fig. S3A at *JXB* online). *ACT11* was expressed in young pistils from anther development stages 9–12, and was also strongly expressed in mature pollen (Huang *et al.*, 1997). Among the 18 transgenic lines we obtained, no pollen developmental defects were observed, although a decrease in *BRP4* expression was detected in the inflorescences of these transgenic plants (Supplementary Fig. S3D–F). This observation further supported the assertion that the expression of *BRP4* in developing anthers is required for proper pollen development.

### *BRP4* affects the progression of male gametophyte mitosis

**Fig. 4.**
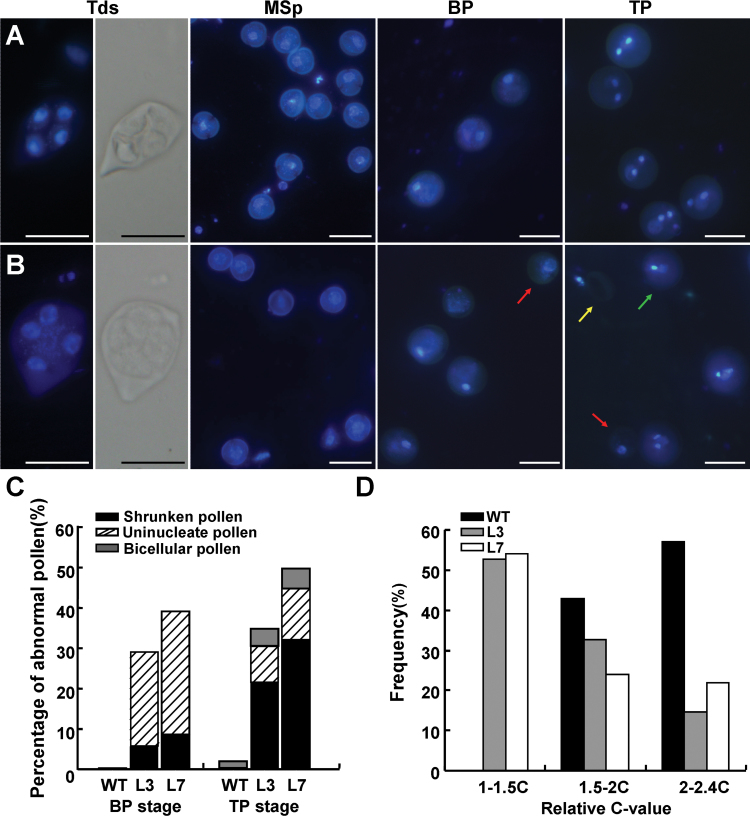
Knockdown of *BRP4* expression arrests the mitotic cell-cycle progression in male gametophytes. (A, B) DAPI staining of tetrads, microspores, and bicellular and tricellular pollen from WT (A) and transgenic *proBRP4:BRP4 RNAi* L7 (B) plants. Note that the abnormal uninucleate pollen (red arrows) and bicellular-like pollen (green arrow) are still present at the bicellular and tricellular development stages in transgenic *proBRP4:BRP4 RNAi* plants. The yellow arrow indicates shrunken pollen. Bar, 20 µm. Tds, tetrads; MSp, microspores; BP, bicellular pollen; TP, tricellular pollen. (C) Quantification of different types of abnormally developed pollen from WT and transgenic *proBRP4:BRP4 RNAi* plants (L3 and L7). BP, bicellular pollen, TP, tricellular pollen. At the BP stage, about 1000 pollen grains were examined, and approximately 2500 pollen grains were examined at the TP stage. (D) Distribution of the DNA content of generative-cell nuclei at prophase from WT bicellular pollen (*n*=42) and generative-like cell nuclei at anthesis in *proBRP4:BRP4 RNAi* L3 (*n*=55) and L7 (*n*=50). The relative C-value of each nucleus was determined by the DAPI fluorescence value normalized against the mean fluorescence of WT nuclei (DNA=2C).

To explore the role of *BRP4* during male gametogenesis, we carefully compared the microspore formation and development of WT and T3 *proBRP4:BRP4 RNAi* transgenic plants. At the tetrad and microspore stages, the male gametophytes appeared to be morphologically normal in both genotypes (Fig. 4A, B). However, a striking difference between the two genotypes was observed starting from the bicellular stage. At this stage, 99.56% of the microspores in the WT had two cells, in which the small generative cell had an intense fluorescent signal but the large vegetative cell had a faint signal (Fig. 4A, C). However, 39.15 and 29.29% of the microspores had only one cell with faint fluorescence in the *proBRP4:BRP4 RNAi* L7 and L3 plants, respectively (Fig. 4B, C), demonstrating that these microspores failed to go through PMI. At the tricellular stage, the percentages of aberrant microspores or bicellular-like pollen in the *proBRP4:BRP4 RNAi* L7 and L3 plants reached 49.74 and 35.07%, respectively. This implies that there are an additional 10.59 and 5.78% of bicellular-like pollen that is incapable of undergoing PMII in the L7 and L3 plants, respectively (Fig. 4A–C). These observations demonstrated that knockdown of *BRP4* expression had no effect on microspore formation but impaired the progression of both PMI and PMII during male gametogenesis.

It was reported previously that *Arabidopsis* generative cells at prophase contain about 2C of the mean DNA content (Friedman, 1999). We conducted DNA content analysis of the generative-like cells of *proBRP4:BRP4 RNAi* L7 and L3 plants at anthesis. In WT generative cells, the distribution of the DNA content ranged from 1.5 to 2.4C, which reflects technical variations in the measurement of fluorescence (Brownfield *et al.*, 2009b). By contrast, more than 50% of the generative-like cells from L7 and L3 had a DNA content of 1–1.5C (Fig. 4D), suggesting that these generative-like cells may be arrested at the G1/S phase during the mitotic cell-cycle progression.

### *ORC6* acts downstream of *BRP4* during male gametogenesis

**Fig. 5.**
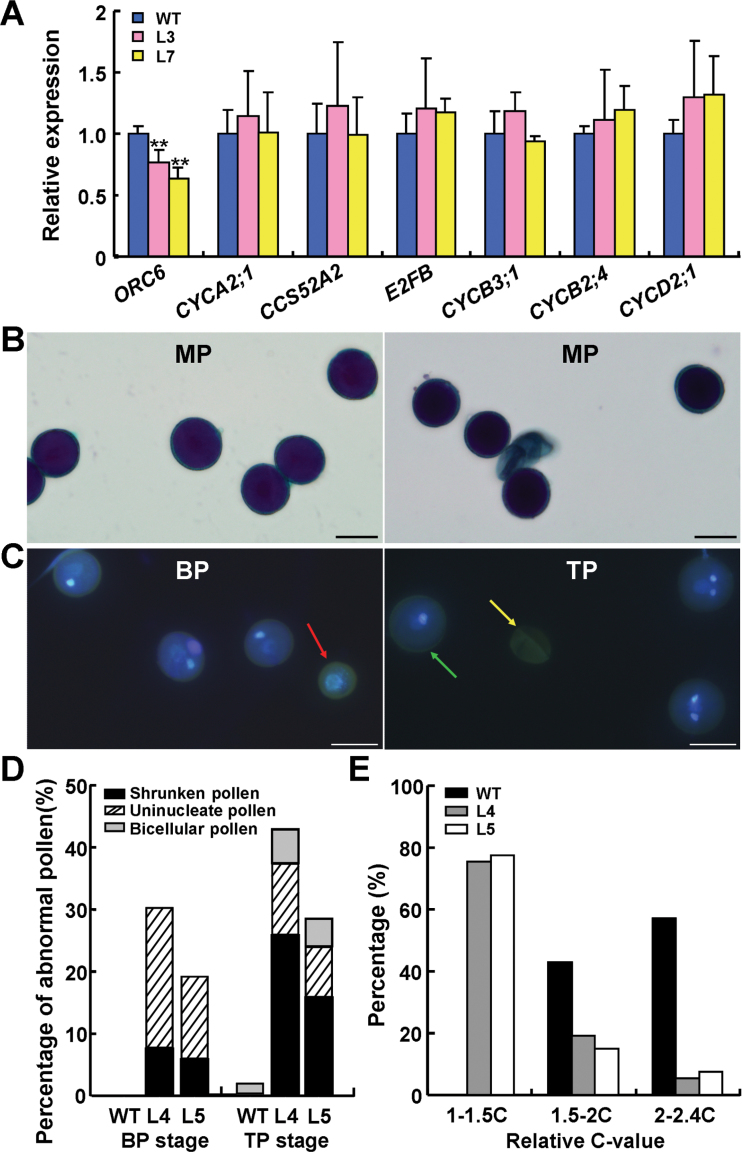
*ORC6* acts downstream of *BRP4* during male gametogenesis. (A) qRT-PCR analysis of cell division-related genes in WT and transgenic *proBRP4:BRP4 RNAi* inflorescences. The data are from three biological replicates and are presented as means±SD (Student’s *t*-test, **P<0.01). Note the decreased expression of *ORC6* in the L3 and L7 plants. (B) Alexander staining of mature pollen (MP) from WT and transgenic *proBRP4:ORC6 RNAi* plants. Bars, 20 µm. (C) DAPI staining of pollen at the bicellular and tricellular stages from transgenic *proBRP4:ORC6 RNAi* anthers. The red arrow shows abnormal uninucleate pollen, the green arrow shows bicellular-like pollen, and the yellow arrow shows shrunken pollen with no DAPI staining. Bars, 20 µm. (D) Percentage of abnormal pollen in transgenic *proBRP4:ORC6 RNAi* L4 and L5 anthers. BP, bicellular pollen; TP, tricellular pollen. At the BP stage, about 1500 pollen grains were examined, and approximately 2500 pollen grains were examined at the TP stage. (E) DNA content analysis of WT generative-cell nuclei at prophase (*n*=42) and undivided generative-like cell nuclei at anthesis in *proBRP4:ORC6 RNAi* L4 (*n*=57) and L5 (*n*=53).

As *BRP4* affects the mitotic cell-cycle progression of male gametophytes, we attempted to identify genes that may function downstream of *BRP4*. We examined expression of genes reported previously to be involved in the regulation of male gametogenesis, *DUO1*, *DUO3*, *RHF1a*, *RHF2a*, and *CDKA;1*, in both WT and *proBRP4:BRP4 RNAi* inflorescences. The expression levels of these genes were similar between the two genotypes (Supplementary Fig. S4A at *JXB* online), implying that these genes might not be downstream of *BRP4*. We then selected seven cell-cycle-related genes whose expression levels were reported to be high during male gametophyte development (Pina *et al.*, 2005), and examined their expression in the inflorescences of both WT and *proBRP4:BRP4 RNAi* plants. As shown in Fig. 5A, the expression of a gene encoding a subunit of the origin recognition complex (ORC), *ORC6*, was obviously decreased in *proBRP4:BRP4 RNAi* inflorescences. The expression levels of the other six genes, *CYCA2;1*, *CCS52A2*, *E2FB*, *CYCB3;1*, *CYCB2;4*, and *CYCD2;1*, were similar between WT and *proBRP4:BRP4 RNAi* plants (Fig. 5A). To validate the possible regulation of *ORC6* by *BRP4*, we generated transgenic plants carrying an oestradiol-inducible *BRP4* construct, *proXVE:BRP4*. After transgenic seedlings were treated with the inducer, *BRP4* was dramatically induced within 4h, and *ORC6* was obviously upregulated after 8h, supporting the notion that *ORC6* functions downstream of *BRP4* (Supplementary Fig. S4B, C).

To investigate the involvement of *ORC6* in *BRP4*-mediated male gametogenesis, we generated transgenic plants harbouring a *BRP4* promoter-driven *ORC6 RNAi* construct (*proBRP4:ORC6 RNAi*) (Supplementary Fig. S5A at *JXB* online). Of the 28 independent transgenic lines examined, 12 displayed pollen developmental defects (Fig. 5B). Detailed examination of the T3 plants of three independent lines (L2, L4, and L5) showed that the percentages of shrunken pollen grains were 36.53, 42.15, and 26.56%, respectively. In contrast, the WT and transgenic plants carrying an empty vector (containing only the *BRP4* promoter fragment) produced 0.67 and 2.2% abnormal pollen, respectively (Supplementary Fig. S5B). Expression analysis showed that the reduced expression of *ORC6* correlated to the percentages of defective pollen in L2, L4, and L5 plants. The expression of other *ORC* genes in these plants did not appear to differ from their expression levels observed in the WT (Supplementary Fig. S5C, D). Further DAPI staining in L4 and L5 revealed that 30.45 and 19.4% of the microspores failed to go through PMI, while another 12.62 and 9.29% were arrested at PMII, respectively (Fig. 5C, D). Moreover, the DNA content analysis of generative-like cells from *proBRP4:ORC6 RNAi* L4 and L5 plants showed that about 75.44 and 77.36% of the aborted generative cells had a DNA content in the range of 1–1.5C (Fig. 4E), suggesting that these generative-like cells are also impaired at the G1/S phase. These observations suggested that *ORC6* acts downstream of *BRP4*, and that it is, at least in part, involved in the *BRP4*-mediated mitotic cell-cycle progression.

## Discussion

### *BRP4* participates in mitotic cell-cycle progression during male gametogenesis

In flowering plants, the two rounds of mitotic division during male gametogenesis are not only involved in cell fate determination but are also required for double fertilization. Several genes that participate in the regulation of male gametophyte development in *Arabidopsis* have been identified recently; these include *DUO1*, *DUO3*, *RHF1a*, *RHF2a*, *FBL17*, *CDKA;1*, and *APD1-4*. These discoveries have improved our understanding of male gametogenesis, yet the molecular basis of this process has not been fully characterized. Here, we showed that the BRP4, a member of the TFIIB-related protein family, is involved in regulation of the mitotic cell-cycle progression during male gametogenesis. Although the gain-of-function mutant of *BRP4*, *smo3-D*, was initially isolated due to its small aerial organ phenotype, our subsequent characterization studies clearly demonstrated that *BRP4* performs a critically important function in mitotic cell-cycle progression during male gametogenesis. *BRP4* is predominantly expressed in male gametophytes at anther development stages 6–8, when microspores are forming and then being released. Transgenic plants harbouring a native promoter-driven *BRP4 RNAi* construct displayed a pollen developmental defect. Moreover, our investigations suggested that this pollen defect resulted primarily from the impaired cell-cycle progression before and after PMI, which led to the production of underdeveloped pollen that subsequently aborted. Therefore, our findings show that *BRP4* plays an important role in the mitotic cell-cycle progression during male gametogenesis.

### BRP4 may function distinctly from other TFIIB-related members

In *Arabidopsis*, the TFIIB subfamily has eight members: TFIIB1, TFIIB2, and six BRP proteins (Knutson, 2013). However, BRP4 appears to be unique member among the TFIIB subfamily proteins of *Arabidopsis*. BRP4 has a long B-finger and B-linker domain at its N terminus, which may be important for the selection of transcription start sites and promoter opening (Kostrewa *et al.*, 2009). Our observation that only transgenic plants overexpressing *BRP4* exhibited the small aerial organ phenotype also supports the notion that BRP4 may mediate a biological process distinct from the other TFIIB subfamily proteins of *Arabidopsis*. Moreover, unlike the expression of the other *TFIIB* genes, which are ubiquitous or restricted to the reproductive organs and seeds, *BRP4* is expressed predominately in developing male gametophytes and in the tapetum from anther development stages 6–8 and in mature pollen. The abundant *BRP4* expression in developing pollen was reported previously in an expression profile analysis (Honys and Twell, 2004). Our observations strongly suggest that *BRP4* plays important roles in male mitotic cell-cycle progression. Additionally, previous transcriptome analysis of anther development stages 4–7 showed that both *BRP4* and *TFIIB1* are expressed at these stages, but only *BRP4* was obviously downregulated in the *sporocyteless* (*spl*) and *excess male sporocytes1* (*ems1*) mutants. *BRP4* was designated previously as a reproductive preferential gene, implying that *BRP4* may be involved in EMS-mediated anther development (Ma *et al.*, 2012).

### *BRP4* affects male gametogenesis, possibly by influencing the cell-cycle progression

In *Arabidopsis*, most of the identified factors involved in male gametogenesis affect the cell division progression of either microspore or generative cells. Our work demonstrated that *BRP4* participates in the regulation of cell-cycle progression in both microspores and generative cells. Surprisingly, the expression levels of genes known to be involved in the mitotic cell-cycle progression of male gametophytes were comparable in the inflorescences of WT and *proBRP4:BRP4 RNAi* plants, suggesting that the role of *BRP4* in regulation of mitotic cell-cycle progression is not dependent on the expression of these genes. Consistent with this, our further analysis indicated that *ORC6* might be downstream of *BRP4*, and that the knockdown of *ORC6* expression in *Arabidopsis* phenocopied the mitotic defects observed in *BRP4 RNAi* male gametophytes. This implied that *ORC6* acts downstream of *BRP4*, and, at least in part, participates in *BRP4*-mediated male gametophyte development. *ORC6* encodes a subunit of the ORC, which is required for the initiation of eukaryotic DNA replication, alterations to chromatin structure, and transcriptional silencing (Bell, 2002; Collinge *et al.*, 2004). *ORC6* expression peaks at the G1/S phase (Diaz-Trivino *et al.*, 2005). Consistently, our DNA content analyses of generative-like cells in both *proBRP4:BRP4 RNAi* and *proBRP4:ORC6 RNAi* plants support the notion that DNA synthesis is impaired in these cells. Therefore, it is likely that *BRP4* participates in the regulation of mitotic cell-cycle progression of male gametophytes, at least in part, through the *ORC6*-mediated cell-cycle regulatory machinery.

## Supplementary data

Supplementary data are available at *JXB* online.

Supplementary Fig. S1. Alignment and phylogenetic analysis of BRP4 and TFIIB-related proteins.

Supplementary Fig. S2. Expression pattern and subcellular localization of BRP4.

Supplementary Fig. S3. Characterization of transgenic *proBRP4:BRP4 RNAi* and *proACT11:BRP4 RNAi* plants.

Supplementary Fig. S4. Identification of genes downstream of BRP4.

Supplementary Fig. S5. Generation and characterization of transgenic *proBRP4:ORC6 RNAi* plants.

Supplementary Table S1. The primers used for expression analysis in this study.

Supplementary Table S2. The primers used for the generation of DNA constructs in this study.

Supplementary Data

## Abbreviations:

DAPI: 4′,6-diamidine-2-phenylindole dihydrochloride
GFP: green fluorescent protein
GUS: β-glucuronidase
ORC: origin recognition complex
PM: pollen mitosis
PIC: pre-initiation complex
RNAi: RNA interference
RT-PCR: reverse transcription-PCR
qRT-PCR: quantitative RT-PCR
SD: standard deviation
TBP: TATA box-binding protein
TAIR: The Arabidopsis Information Resource
WT: wild type.
