# Supplementary material for: The Arabidopsis transcription factor IIB-related protein BRP4 is involved in the regulation of mitotic cell-cycle progression during male gametogenesis
Source: J Exp Bot. 2014 Apr 10;65(9):2521–31. doi: 10.1093/jxb/eru140 (PMC4036515; doi:10.1093/jxb/eru140)
Supplement: Supplementary Data [file supp_65_9_2521__index.html]

The Arabidopsis transcription factor IIB-related protein BRP4 is involved in the regulation of mitotic cell-cycle progression during male gametogenesis — The Arabidopsis transcription factor IIB-related protein BRP4 is involved in the regulation of mitotic cell-cycle progression during male gametogenesis — Supplementary Data 

# The *Arabidopsis* transcription factor IIB-related protein BRP4 is involved in the regulation of mitotic cell-cycle progression during male gametogenesis

## Supplementary Data

Data files

**Files in this Data Supplement:**

- Supplementary Data - Supplementary Data
