# Supplementary material for: The Arabidopsis transcription factor IIB-related protein BRP4 is involved in the regulation of mitotic cell-cycle progression during male gametogenesis
Source: J Exp Bot. 2014 Apr 10;65(9):2521–31. doi: 10.1093/jxb/eru140 (PMC4036515; doi:10.1093/jxb/eru140)
Supplement: Supplementary Data [file supp_eru140_jexbot113563_file001.pdf]

The *Arabidopsis* TFIIB-related protein BRP4 is involved in the regulation of mitotic cell cycle progression during male gametogenesis

Zhixiang Qin, Xiaoran Zhang, Xiao Zhang, Wei Xin, Jia Li and Yuxin Hu*

Supplementary Data:

Supplementary Fig. S1-S5

Supplementary Table S1-S2

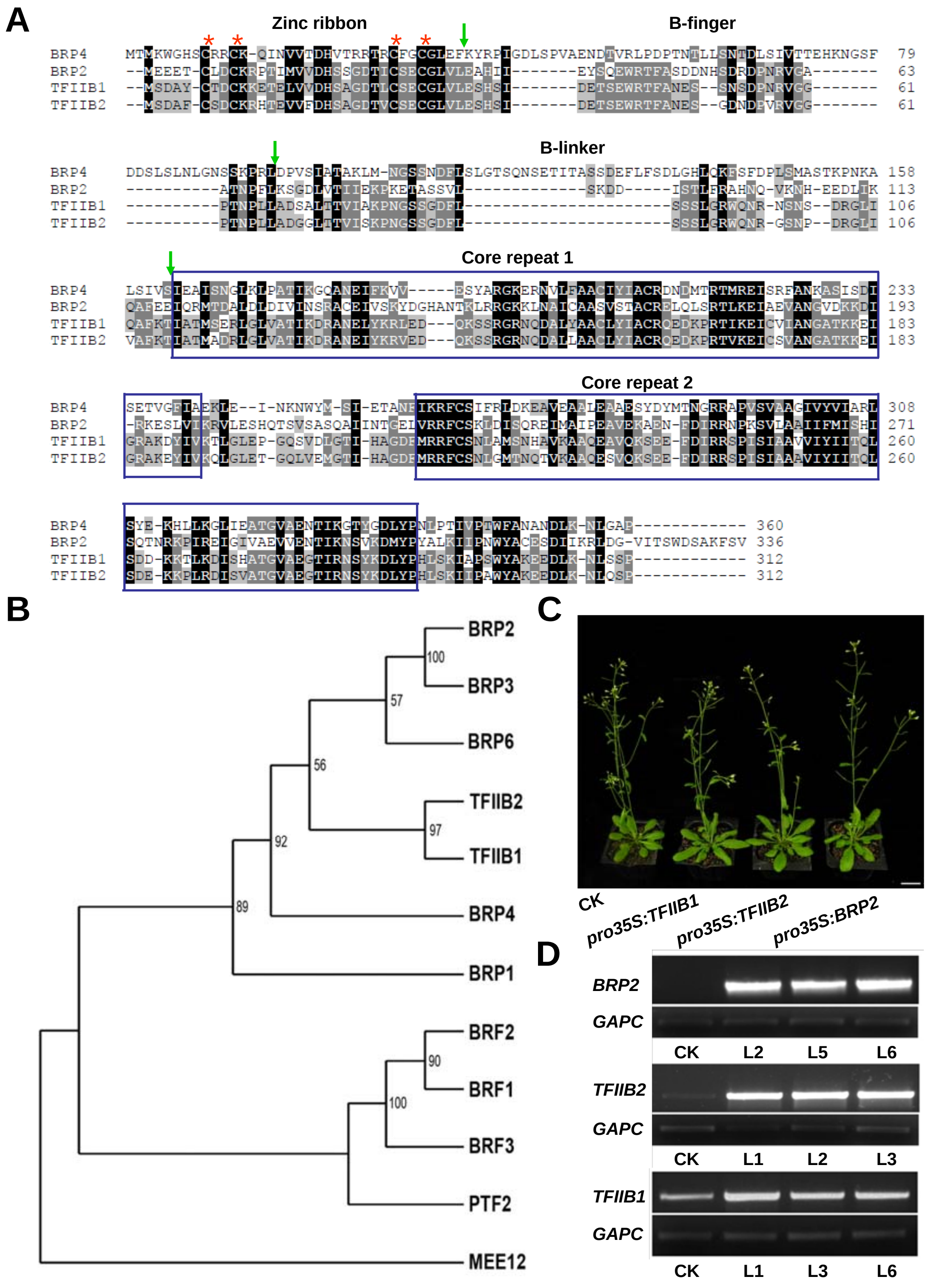

Supplementary Fig. S1. Alignment and phylogenetic analysis of BRP4 and TFIIB-related proteins.

(A) Sequence alignment of BRP4 with its close related homologues. Red stars refer to the Cys residues for zinc ribbon domain and green arrows to the borders of B-finger and B-linker domains. The blue boxes show the two core repeat domains. (B) Phylogenetic analysis of *Arabidopsis* TFB-like proteins. (C) Morphology of forty-day-old transgenic plants carrying an empty vector (CK), *pro35S:TFIIB1*, *pro35S:TFIIB2*, *pro35S:BRP2* construct, respectively. Scale bar: 2 cm. (D) Expression of *TFIIB1*, *TFIIB2*, *BRP2* in CK and their three independent transgenic plants (as L1 to L6), respectively.

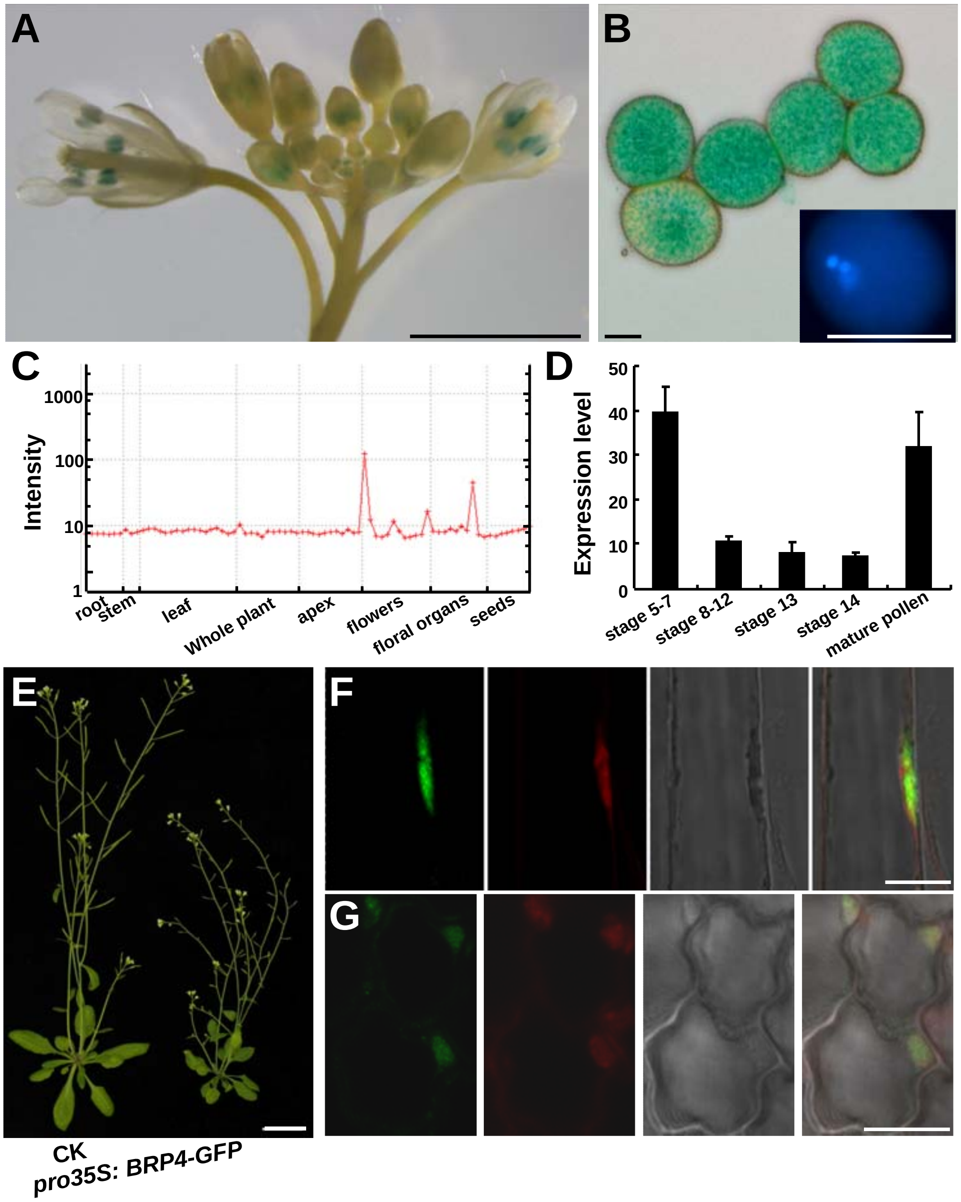

Supplementary Fig. S2. Expression pattern and subcellular localization of BRP4.

(A) GUS staining of transgenic inflorescence carrying a *proBRP4:GUS* construct. Scale bar: 3mm. (B) GUS and DAPI staining of *proBRP4: GUS* mature pollen. Scale bar: 20μm. (C) Expression profile of *BRP4* in different organs revealed by AtGenExpress Visualization Tool (AVT) in TAIR database. (D) Expression level of *BRP4* in flowers at anther development stage 5-14 and in mature pollen revealed by *Arabidopsis* electronic Fluorescent Pictograph (eFP) browser in TAIR database. (E) Morphology of forty-day-old seedlings of CK and *pro35S: BRP4-GFP* plants. Scale bar: 2 cm. (F-G) Nucleus localization of BRP4-GFP protein in root (F) and petal (G) cells. Root and petal epidermal cells of transgenic plants carrying a *pro35S: BRP4-GFP* construct were used to determine fusion protein localization. The GFP signal, DAPI staining, bright-field and overlaid images are shown from left to right. Scale bar: 10 μm.

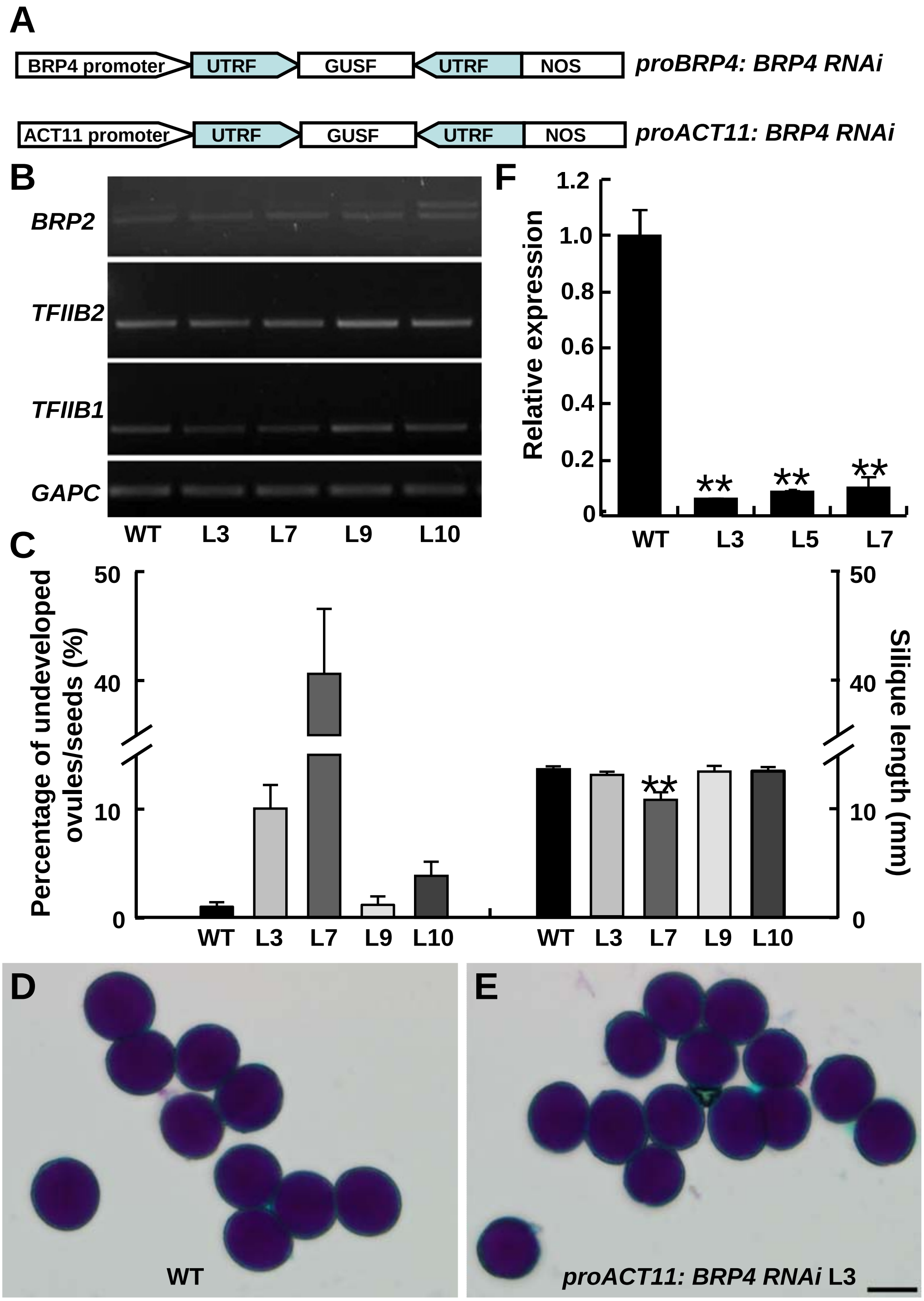

Supplementary Fig. S3. Characterization of transgenic *proBRP4:BRP4 RNAi* and *proACT11:BRP4 RNAi* plants.

(A) Schematic illustration of *proBRP4:BRP4 RNAi* and *proACT11:BRP4 RNAi* constructs. (B) Expression analysis of *TFIIB1*, *TFIIB2* and *BRP2* in WT and transgenic *proBRP4:BRP4 RNAi* inflorescences (L3, L7, L9 and L10). (C) Fertility analysis of WT and *proBRP4:BRP4 RNAi* siliques as described in (B). Sixteen siliques from eight plants were measured for each line and data are shown as mean values±SD (Student's *t*-test, **P<0.01). (D-E) Alexander's staining of mature pollen in WT (D) and *proACT11:BRP4 RNAi* plants (E). Scale bar: 20 µm. (F) Expression of *BRP4* in transgenic *proACT11:BRP4 RNAi* inflorescences. The data are from three biological replicates and shown as mean values±SD (Student's *t*-test, **P<0.01).

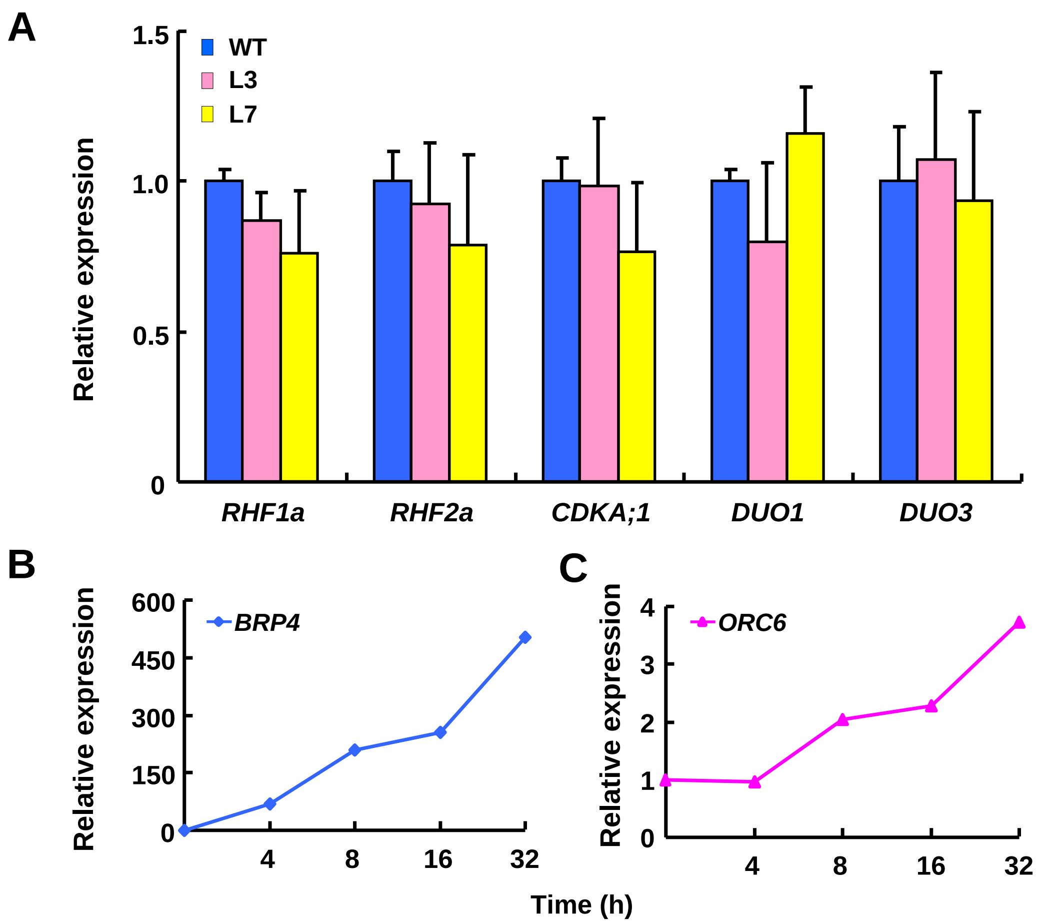

Supplementary Fig. S4. Identification of genes downstream of *BRP4*.

(A) Expression analysis of genes involved in mitotic cell cycle progression of male gametophytes in WT and transgenic *proBRP4:BRP4 RNAi* plants. (B-C) *ORC6* is up-regulated by expression of *BRP4*. The transgenic seedlings harboring a chemical-inducible *proXVE:BRP4* construct were treated with 10 µm (+)17-ß-estradiol for times indicated, and expression levels of *BRP4* (B) and *ORC6* (C) were determined by qRT-RCR. The data are from three biological replicates.

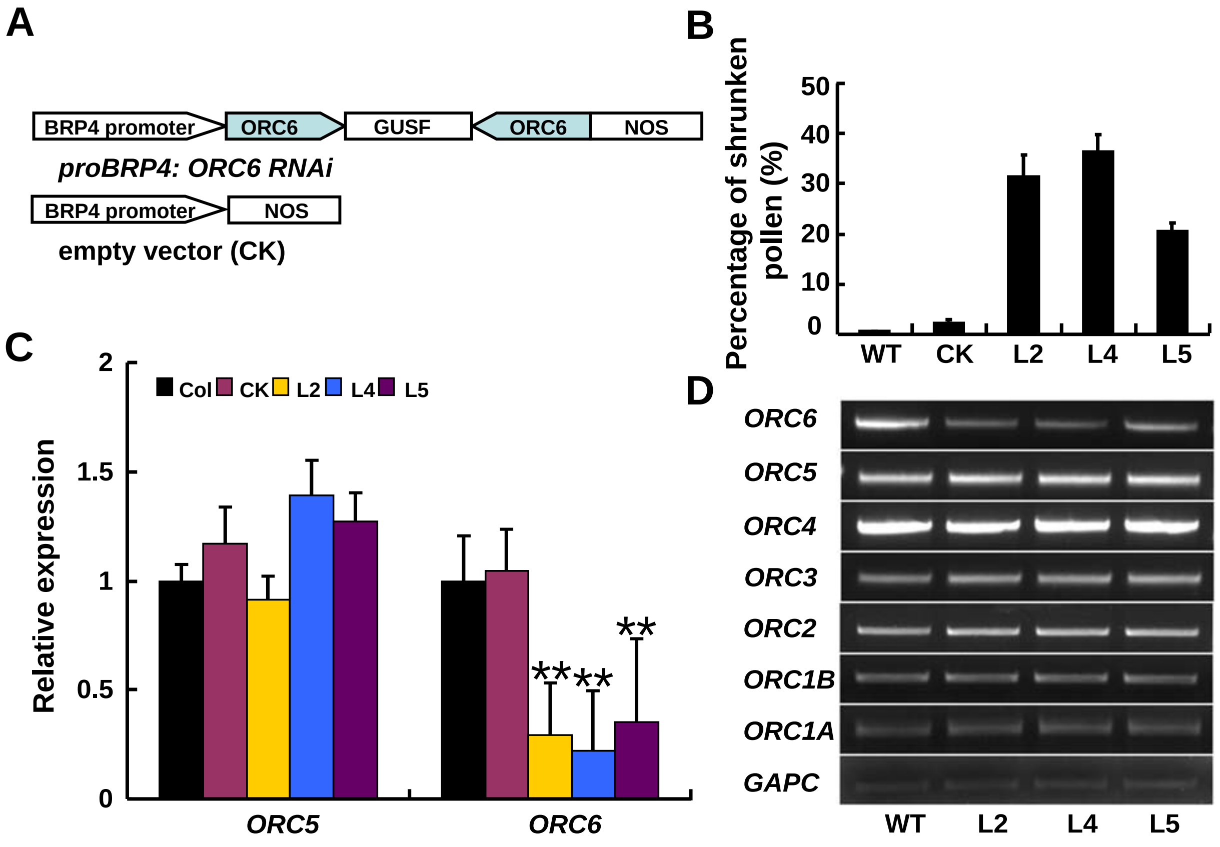

Supplementary Fig. S5. Generation and characterization of transgenic *proBRP4:ORC6 RNAi* plants.

(A) Schematic illustration of *proBRP4:ORC6 RNAi* construct and an empty vector containing only the *BRP4* promoter (CK). (B) Percentages of the shrunken pollen in WT, CK and transgenic *proBRP4:ORC6 RNAi* L2, L4 and L5 plants. (C) Expression of *ORC5* and *ORC6* in WT, CK and transgenic *proBRP4:ORC6 RNAi* lines as described in (B). The data are from three biological replicates and shown as mean values±SD (Student's *t*-test, **P<0.01). (D) RT-PCR analysis of *ORC* genes in WT and transgenic *proBRP4:ORC6 RNAi* lines as described in (B).

Supplementary Table S1. The primers used for expression analysis in this study

Primers	Sequences (5'-3')	Description
BRP4-QF	TGTCACCACCGAGCACAAGAATG	qRT-PCR
BRP4-QR	TTCATCGGAAGAAGCAGTAATCGTC	
BRP4-insiF	GCGACTCCATTGAAAGAAGA	In-situ
BRP4-insiR	TCAAGACCACACCCGAAG	
RHF1a-QF	TCTTTATCGTCTCTCTGCTATTTCCCA	qRT-PCR
RHF1a-QR	CGAGGATGCTTCAGGTGATTTTG	
RHF2a-QF	TACAACATCTTCCAGTGGGGGTG	qRT-PCR
RHF2a-QR	ATGTCCTTGACTTGACGACCTGC	
DUO1-QF	TTAAGTTAGAGCATCAGCCTTTCGC	qRT-PCR
DUO1-QR	TGAGGGAGAGCGAACAATGGC	
DUO3-QF	AAGGAGAAGAAGGCTCAGAATGCG	qRT-PCR
DUO3-QR	GCTACTCGGATCCAGACTCAACCAA	
CDKA;1-QF	GATTGGTGAAGGAACTTACGGTGTG	qRT-PCR
CDKA;1-QR	GGAACACCTTCATCCTCCTGCTCTA	
ORC6-QF	CGCCGTCTTTGCGATGCTC	qRT-PCR
ORC6-QR	TCAATTTAACCGCTGCTTGCCTAT	
ORC4-F	CGGCGGAGAAATCCCTAAATC	RT-PCR
ORC4-R	CTTTGCTCCTTCACTTCTAACCTCC	
ORC1A-F	AAAAAGTCAGGTCAAACACTTGTGG	RT-PCR
ORC1A-R	TCGAAACCCCACGAACACTCT	
ORC1B-F	AAAAAGTCTGGTCAAAGTCAAACA	RT-PCR
ORC1B-R	TCAAACCTCCTCGCTTACTCTTCAA	
ORC2-F	ATGGAGGACATTGAGAACATAGAAGA	RT-PCR
ORC2-R	CTACTGATTGAGATCAAGCAAAAGCT	
ORC3-F	TTCACAAGGCTTCGTCTGGTAAT	RT-PCR
ORC3-R	CTCGTCACAGATGGCAAGTCAAA	
ORC5-F	AACTCAGAACCTATTGAATCCCACA	RT-PCR
ORC5-R	GCCATCATGAGGGTGACTAGAGA	
CYCA2;1-QF	AGACCGAGTAATAAACGAATGGCA	qRT-PCR
CYCA2;1-QR	TGCTTAGTTTCTTTACCACCTCGC	
CCS52A2-QF	TGTGAACACGCCGCAGCA	qRT-PCR
CCS52A2-QR	AGCAGTGCCACCACCAGAAG	
E2FB-QF	AACAGGATTCAGTGGAAGGGTCT	qRT-PCR
E2FB-QR	TTTCAGTGACGAACAGTAACCTTTT	
CYCB3;1-QF	GAACACTCTAAGGATTCCAACGCT	qRT-PCR
CYCB3;1-QR	ACCAACTGAGACCCTTTTCGGAT	
CYCB2;4-QF	TTGTGCTGTCAAGAGACCATTCA	qRT-PCR
CYCB2;4-QR	CATCTGTTATGATTCTATCTAATGCTTCG	
CYCD2;1-QF<br>CYCD2;1-QR	AACTATGGCGGCGGATTTACGA<br>CGTTGCCGCCAAAGTTGTCGT	qRT-PCR
57380-F	GAAAGCACCATACCCCAGGTT	RT-PCR
57380-R	TATTCCCAAGCATCAACGATGATTC	
ORC5-QF	CTCAGAACCTATTGAATCCCACA	qRT-PCR
ORC5-QR	AAGAGTAGGGCAATCTAACGGC	
ORC6-R	TCTTTGGTCTCAGATTTCTTTGGGAA	RT-PCR with ORC6-QF

Supplementary Table S2. The primers used for the generation of DNA constructs in this study

Primers	Sequences (5'-3')	Description
57370F	ATGACGATGAAGTGGGGTCACA	*pro35S:antiBRP4* construct and RT-PCR
57370R	CTAAGGAGCTCCAAGGTTTTTCAG	
57370-SF	ctcgagccATGACGATGAAGTGGGGT	*pro35S:BRP4* construct
57370-SR	aagcttCTAAGGAGCTCCAAGGTTTTTC	
BRP2F	ggatccATGGAAGAAGAGACCTGCTTGGAC	*pro35S:BRP2* construct and RT-PCR
BRP2R	gaattcTTATACTGAAAATTTTGCAGAATCCCA	
TFIIB1F	ggatccATGTCGGATGCGTATTGTACGG	*pro35S:TFIIB1* construct and RT-PCR
TFIIB1R	gtcgacTCAAGGACTTGACAGGTTTTTCAGA	
TFIIB2F	gtcgacATGAGTGACGCGTTTTGTTCGG	*pro35S:TFIIB2* construct and RT-PCR
TFIIB2R	gaattcTCAAGGGCTTTGAAGGTTCTTGA	
BRP4-AscR	ggcgcgccaAGGAGCTCCAAGGTTT	*pro35S:BRP4-GFP* construct with 57370F
ACT11F	gaattcTCATATCTTCTGCTTTTCTGT	*proACT11:BRP4 RNAi* construct-promoter fragment
ACT11R	ggtaccTTTCTATATCCTGTCAAAATT	
BRP4i-F	gagctcGTGTTCTCTTGTCGCCGTTA	*proBRP4:BRP4 RNAi* construct-promoter fragment
BRP4i-R	ggtaccCGAAACCCTAATTTTCTGTCC	
BRP4-FF	tctagaGCGACTCCATTGAAAGAAGA	*proBRP4:BRP4 RNAi* and *proACT11:BRP4 RNAi* construct-RNAi fragment
BRP4-FR	ctgcagTCAAGACCACACCCGAAG	
BRP4-RF	ctcgagatctGCGACTCCATTGAAAG	
BRP4-RR	atcgatTCAAGACCACACCCGAAG	
ORC6-FF	tctagaGAGGTTTCTTGCGTCACTACCAG	*proBRP4:ORC6 RNAi* construct-RNAi fragment
ORC6-FR	ctgcagATCCCAACACAGTCAAAACAAAG	
ORC6-RF	ggtaccGAGGTTTCTTGCGTCACTACCAG	
ORC6-RR	ctcgagATCCCAACACAGTCAAAACAAAG	
BRP4-induF	ctcgagATGACGATGAAGTGGGGT	*proXVE:AtBRP4* construct
BRP4-induR	actagtCTAAGGAGCTCCAAGGTT	
BRP4-F	gtcgacAGGAGGATAATCTGATAG	*proBRP4:GUS* construct
BRP4-R	ggatccCGAAACCCTAATTTTCTGTCC	
